# A decade of health research capacity building in Honduras: institutional transformation, challenges, and lessons learned

**DOI:** 10.1080/16549716.2025.2574734

**Published:** 2025-10-30

**Authors:** Gustavo Fontecha, Ana Sánchez, Gabriela Matamoros, Denis Escobar, Bryan Ortiz

**Affiliations:** a Instituto de Investigaciones en Microbiología, Facultad de Ciencias, Universidad Nacional Autónoma de Honduras, Tegucigalpa, Honduras; b Department of Community Health Sciences, Brock University, St. Catharines, Ontario, Canada

**Keywords:** research collaboration, higher education, Central America, international cooperation, research personnel

## Abstract

**Background:**

Honduras has historically faced major barriers to building a sustainable health research system, including minimal R&D investment and limited institutional infrastructure. A Canadian-funded initiative (2007–2012) established the first research-oriented MSc program, a non-clinical ethics board, and modern laboratories at the *Universidad Nacional Autónoma de Honduras* (UNAH).

**Objective:**

This article examines how health research capacity evolved between 2013 and 2025, highlighting long-term outcomes, enablers, and barriers, and situating these within a regional Central American comparison. The narrative, largely anecdotal, reflects on the experience and impact of biomedical research at UNAH, particularly through the *Instituto de Investigaciones en Microbiología* (IIM).

**Methods:**

Alumni trajectories and institutional transformations are illustrated with concrete examples. Bibliometric analysis contextualizes scientific output, complemented by broader indicators (GDP, R&D investment, tertiary education, PhDs per million) from World Bank sources.

**Results:**

More than 30 MSc graduates have strengthened biomedical and public health institutions, with several completing doctoral training abroad and returning to Honduras. Since its formal creation in 2014, the IIM has produced over 170 publications, representing more than 20% of UNAH’s health-related output since 2012. Challenges to sustainability include chronic underinvestment (< 0.1% GDP in R&D), rigid bureaucracy, limited career pathways, and brain drain. Enablers have been international partnerships, the academic diaspora, and strong local leadership.

**Conclusion:**

The Honduran case illustrates how targeted, multi-level investment in individuals, institutions, and governance can foster long-term research capacity in resource-constrained settings, while underscoring the need for national policies, career structures, private sector engagement, and sustained international collaboration.

## Paper context

- **Main findings**: Honduras has expanded its scientific capacity through graduate training, institutional strengthening, and international collaboration, despite persistent structural and financial constraints.
- **Added knowledge**: This analysis provides the first longitudinal account of how local leadership, returning scientists, and partnerships have gradually shaped a national research environment in Honduras.
- **Global health impact for policy and action**: The Honduran experience highlights that sustained investment and coordinated strategies can foster research systems in low-resource settings, offering lessons for strengthening scientific capacity across Central America and other comparable regions.

## Background

In 2013, Sánchez and colleagues reported in *Global Health Action* the initial outcomes of a Canadian-funded initiative to strengthen health research capacity in Honduras between 2007 and 2012 [1]. At the time, the country lacked a national health research system, invested only 0.04% of its GDP in science and technology [2], among the lowest in Latin America, and had virtually no scientific output. The initiative led to the creation and implementation in 2009 of the first Research-oriented MSc program in infectious and zoonotic diseases at the UNAH, along with the establishment of a non-clinical research ethics board, laboratory infrastructure, and a biosafety training center, key steps toward building a sustainable research culture at the university.

The initiative led by Sanchez et al. was driven by the conviction that strengthening health research capacity in low- and lower-middle-income countries is essential – not only to address the heavy disease burden with limited resources, but also to generate locally relevant evidence that can inform more effective and context-sensitive health policies [3,4]. Moreover, the development of a robust research infrastructure enabled active participation in global health dialogues and fostered the production of scientific knowledge with regional significance.

More than a decade after the completion of the Canadian-funded initiative, this article critically examines how research capacity has evolved at UNAH, focusing on institutional outcomes, challenges, and enabling factors that have shaped sustainability. To situate Honduras within the broader regional landscape, a comparison is provided with the leading public universities of the five other Central American countries, based on publication output. The analysis underscores enablers and barriers specific to Honduras and draws lessons that may inform stakeholders seeking to strengthen research capacity in resource-limited settings.

## Methods

The primary sources of information were institutional records from UNAH, which provided data on MSc graduates, the creation of auxiliary researcher positions, and research outputs, complemented by annual reports from the IIM. Alumni trajectories were reconstructed through direct communication and institutional follow-up, documenting postgraduate studies abroad, return rates, and current employment.

To situate the Honduran case within a broader regional perspective, a bibliometric analysis was conducted using the Scopus (Elsevier) database [5]. Searches were conducted between July and August 2025, covering 2000–2025. Searches were filtered by the institutional affiliation of the six leading public universities in Central America – Universidad de San Carlos de Guatemala, Universidad de El Salvador, Universidad Nacional Autónoma de Honduras, Universidad Nacional Autónoma de Nicaragua en León, Universidad de Costa Rica, and Universidad de Panamá—and included both English- and Spanish-language outputs. For each university, all indexed publications were tabulated, and health-related outputs were identified using Scopus subject area classifiers: Agricultural and Biological Sciences; Medicine; Biochemistry, Genetics and Molecular Biology; Immunology and Microbiology; Nursing; Neuroscience; Dentistry; and Veterinary.

Finally, contextual indicators such as gross domestic product (GDP) [6], national investment in research and development (R&D) [7], number of researchers in R&D per million inhabitants [8], were retrieved from World Bank sources to provide background context and facilitate regional comparisons of research capacity.

## Historical background and the 2007–2012 initiative

In the early 2010s, Honduras faced severe structural constraints that hindered the development of a national health research agenda. More than 60% of the population lived in poverty [9], while the country grappled with political instability, exacerbated by the 2009 *coup d’état*, and persistent weaknesses in governance [2]. Honduras lacked a research council or agency to coordinate or fund scientific work, and health research was largely absent from policy agendas [10]. The higher education system mirrored these structural limitations. Public universities, including UNAH, operated under a teaching-centered model that consistently deprioritized research. Academic careers offered neither protected time for research nor structured pathways for advancement, with scarce funding opportunities and limited institutional recognition. Early-career faculty were particularly affected, as research was often undervalued and perceived as disconnected from pressing health needs. The absence of internal grant mechanisms, inadequate laboratory infrastructure, and burdensome bureaucracy further discouraged scientific inquiry [11]. These barriers were compounded by the end of Swedish development cooperation (Sida) through the Karolinska Institute Research Training (KIRT) program, which from 1988 to 2012 had trained 11 Honduran microbiologists [12]. The withdrawal of Swedish cooperation left a significant gap in institutional strengthening at UNAH, disrupting scientific collaboration, graduate training, and research infrastructure.

Against this backdrop, Canada’s Global Health Research Initiative (CIHR, IDRC, and other partners) launched a five-year project in 2007 to strengthen research capacity at UNAH. Documented by Sánchez et al. [1,13], it addressed training and systemic barriers. Key components included an MSc in infectious and zoonotic diseases; a state-of-the-art lab; UNAH’s first non-clinical Research Ethics Board; a biosafety training center; and support for regional and global networks. The project adopted a multi-level model targeting human resources, institutions, governance, and partnerships. Crucially, Honduran faculty were trained to lead academic and administrative aspects, ensuring national alignment and sustainability. By 2012, early impacts were visible: the MSc program had graduated its first cohort; the ethics board was operational; and researchers were publishing, securing external funding, and joining regional networks. These marked the emergence of a nascent research ecosystem at UNAH. The following findings explore how these gains evolved over the next 13 years and identified the factors that supported their sustainability.

## Capacity building outcomes (2013–2025)

### Highly qualified personnel (HQP)

One of the most tangible outcomes of the 2007–2012 initiative has been the steady growth of trained professionals in Honduras’s biomedical research sector. According to UNAH records, the MSc program in infectious and zoonotic diseases has graduated over 30 students across four cohorts. While modest compared to countries with established postgraduate systems, this represents a remarkable achievement in Honduras, where academic postgraduate training previously required studying abroad. In the absence of local doctoral programs in biomedical sciences, several MSc graduates have pursued PhDs overseas, with some returning to strengthen national academic and public health institutions. At UNAH, a few have joined the Faculty of Sciences, reinforcing research and teaching in microbiology, parasitology, and public health, and helping to bridge the gap between teaching and research. MSc graduates and other faculty have also benefited from international exchanges, most notably through the German Academic Exchange Service (DAAD) [14], which continues to provide valuable opportunities for training, collaboration, and global networking. Beyond academia, several MSc graduates have joined public health institutions and diagnostic laboratories. Their work has reinforced disease surveillance, outbreak response, and diagnostics, strengthening the country’s ability to address health threats such as dengue, COVID-19, and antimicrobial resistance.

At the same time, numerous undergraduates have gained access to research facilities, often experiencing the scientific method for the first time. These opportunities have provided hands-on training and, in some cases, milestones such as contributing to publications or mastering advanced techniques, giving students more competitive profiles for graduate studies abroad. In a country with very few researchers per capita, the cumulative impact of these experiences has been significant. To further professionalize research careers, UNAH established in 2015 the position of auxiliary researcher, a full-time post for professionals with a master’s or doctoral degree. Between 2014 and 2022, a total of four positions were created at UNAH. These posts are full-time and formally tenured. These researchers lead original studies, supervise students, and contribute to academic output, while being exempt from undergraduate teaching to dedicate themselves fully to research. However, despite its initial promise, the role has received limited institutional support, its expansion has stalled, and successive administrations have failed to strengthen this successful model.

### Institutional strengthening

While increases in HQP have been notable, institutional sustainability remains a key challenge. Nonetheless, one of the most significant structural achievements has been the creation of the IIM at UNAH [15]. This research institute, with formal university recognition and autonomous governance, now includes four research laboratories. The IIM operates under a diversified funding model, combining institutional support, competitive external grants (e.g. Namru-South, Médecins Sans Frontières, CSUCA), and income from diagnostic and technical services. This has enabled advanced resources previously unavailable in Honduran academia. Additionally, four microbiology research groups, composed of UNAH faculty and external collaborators, are affiliated with the IIM [15]. Institutional strengthening has also been supported by the sustained operation of the Research Ethics Board established during the original project. This board now serves multiple faculties and research groups and is recognized nationally for its leadership in biomedical research ethics.

### Scientific production and visibility

**Figure 1.**
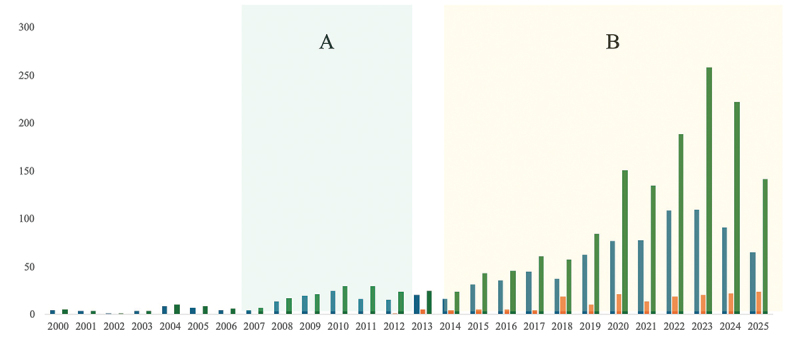
Annual number of total publications (green) and health-related publications (blue) from UNAH, compared with publications from the IIM (orange). Period A corresponds to the years of the Canadian cooperation project for research capacity building, while period B represents the post-initiative period following the establishment of UNAH’s first research center.

Although Honduras’ overall scientific output remains modest, there has been clear progress in both quality and visibility, particularly within research groups strengthened through capacity-building initiatives. The IIM, a relatively new unit with only a handful of researchers, has emerged as one of the top contributors to peer-reviewed publications both within UNAH and at the national level. Since 2012, its researchers have co-authored more than 170 articles indexed in Scopus [15]. This output represents 11.7% of the university’s total publications between 2012 and August 2025, and 21.63% of all publications in the health sciences during that period (Figure 1).

### Research engagement and collaborative networks

Between 2012 and 2025, the IIM has expanded its participation in regional and international research projects. These include studies on vector-borne diseases [16–18], malaria surveillance and resistance mapping [19–33], One Health initiatives addressing leishmaniasis [34–41] and geohelminths [42–48], and investigations into emerging fungal pathogens [49–58], among others. Through these projects, Honduran scientists have accessed advanced training, and contributed to regional and global research agendas. Despite ongoing systemic challenges, these achievements highlight the value of long-term capacity-building investments when supported by institutional commitment and regional collaboration.

## Regional background comparison: research capacity in Central American national universities

To contextualize UNAH’s recent progress, it is important to situate Honduras within the broader landscape of Central American higher education and research. Comparing scientific output and structural indicators across the region’s leading public universities highlights both shared challenges and significant disparities in research capacity. This perspective is not intended as a ranking exercise, but rather to emphasize structural factors – such as R&D investment, research workforce availability, and institutional environments – that determine the ability of universities to sustain scientific activity. By contrasting UNAH’s trajectory with that of its regional peers, we can better understand the opportunities and constraints shaping research development in Honduras.

### Indexed scientific publications in health

Per capita publication output in Central America remains below the global average [59]. Scopus data [5] show that, when comparing total scientific output and health-related publications from the region’s leading public universities, the UCR stands out by a wide margin, with over 11,000 total publications including about 7,000 in health from 2000 to 2025. The UP and UNAH follow at a much smaller scale, each with under 2,000 total, including 1,500 health-related publications. The USAC, UNAN-León, and UES lag further behind, all with fewer than 1,000. Overall, UCR dominates regional research output, underscoring persistent disparities in research capacity (Table 1).

**Table 1.** Comparative indicators of research and higher education capacity in six Central American countries. Data include GDP per capita (USD, 2024), percentage of GDP invested in research and development (R&D), number of researchers in R&D per million people, and bibliometric outputs indexed in Scopus (2000–2025). Publication counts are filtered by institutional affiliation with each country’s leading public university, with a distinction between total indexed publications and those classified as health-related.

Country	GDP per capita (USD, 2024) [6]	% GDP in R&D (most recent year) [7]	Researchers in R&D per million people (most recent year) [8]	Total indexed publications (2000–2025) affiliated with the country’s leading public university [5]	Health-related publications (2000–2025) affiliated with the country’s leading public university [5]
Costa Rica	18 587	0.34%	462	11 923	6 919
Panamá	19 102	0.16%	253	1 822	1 249
Honduras	3 426	0.06%	188	1 591	892
Guatemala	6 150	0.06%	15	1 004	670
Nicaragua	2 847	0.11%	71	500	432
El Salvador	5 579	0.14%	58	477	300

### Investment in R&D as a percentage of GDP

Experts often recommend that developing countries aim for at least 1% of GDP in R&D spending to begin building a sustainable innovation ecosystem [60]. Across Central America, investment in R&D as a share of GDP remains critically low. World Bank data indicate that Honduras and Guatemala allocate the least to R&D (0.06%), followed by Nicaragua (0.11%), El Salvador (0.14%), and Panama (0.16%) [61]. Such levels are far below the suggested investment and fall short of what is required [62]. In contrast, Costa Rica stands out as the regional exception, with R&D investment reaching approximately 0.34% [61].

### Research workforce and structural disparities

Beyond publication output and financial investment, the availability of human capital dedicated to research further illustrates regional inequities. Costa Rica again leads with 462 researchers per million people, followed by Panama (253) and Honduras (188). In stark contrast, Guatemala, Nicaragua, and El Salvador report fewer than 75 researchers per million, highlighting their limited scientific workforce. These structural gaps help explain the uneven productivity observed in Scopus outputs and reflect broader weaknesses in national research systems. Combined with persistently low R&D spending, such disparities hinder the consolidation of competitive research environments and perpetuate dependence on external collaborations for scientific advancement.

## Factors enabling research ecosystems

### Research ecosystems and opportunities for young scientists

Central American countries such as Costa Rica and Panama have developed structured research systems. For example, Panama, for instance, is home to the century-old Gorgas Memorial Institute [63] whose new headquarters recently received an investment exceeding 200 million USD [64]. Another key driver of Panama’s progress is the *Secretaría Nacional de Ciencia, Tecnología e Innovación* (SENACYT) [65], which plays a central role in promoting and strengthening research. In Costa Rica, the UCR stands out as a leading knowledge-generating institution, hosting more than 70 research centers and institutes. Although Honduras and neighboring countries still contend with fragmented systems and weak governance, efforts at UNAH over the past decade have begun to bear fruit, creating research spaces, graduate training opportunities, and new institutional frameworks. These advances show that progress is possible even in resource-constrained environments. Yet, sustained investment and stronger national support remain essential for consolidating these gains and moving closer to the construction of a fertile and enduring scientific ecosystem at UNAH.

### Strategic role of international partnerships and the academic diaspora

International collaboration has been central to UNAH’s research capacity development. Beyond financial aid, partnerships with Canadian, U.S., European, and Latin American institutions facilitated knowledge exchange, exposure to global standards, and joint research agendas. These collaborations also provided graduate training, fellowships, and co-publication opportunities. The return of Honduran researchers trained abroad, particularly PhD holders supported through international fellowships, has been critical for consolidating research groups and mentoring new scientists. Returnees often act as institutional bridges, bringing grant-writing skills, scientific rigor, and networks that align local research with global standards. At the same time, some Honduran researchers have migrated to countries with more advanced scientific development, yet this ‘brain gain’ has strengthened local capacity through continued collaboration.

### Local leadership and institutional ownership

Progress would not have been possible without committed local leadership. Faculty champions at UNAH played a central role in aligning research with national priorities, securing resources, and embedding scientific activity within institutional frameworks. Initiatives such as the MSc program and the IIM have flourished thanks to the vision and persistence of these leaders, who built a supportive research environment not because of the institution, but despite it. This highlights the critical importance of institutional ownership: locally led initiatives embedded within a university’s strategic plans are far more likely to endure beyond external funding, whereas projects lacking strong internal leadership often fail to achieve long-term sustainability or scale.

## Challenges and barriers to sustainability

Despite meaningful gains in health research capacity over the past two decades, long-term sustainability remains limited by persistent and interrelated challenges at the national, institutional, and systemic levels. These barriers reflect broader structural weaknesses in Honduras’s science and technology ecosystem and continue to restrict the full potential of prior capacity-building efforts.

### Chronic underinvestment in research and development

National spending on R&D has remained below 0.1% of GDP [66], restricting support for research agendas, early-career recruitment, and infrastructure expansion. At UNAH, limited and unstable internal funds are further weakened by inefficient fiscal oversight and bureaucratic delays. As a result, researchers depend on unpredictable external grants or income from service provision – unsustainable strategies for long-term growth.

### Rigid and fragmented university bureaucracy

Procedural rigidity at UNAH continues to hinder research execution. Procurement, recruitment, collaboration, and funding management are slow, and poorly aligned with scientific needs. These inefficiencies raise costs, delay projects, and discourage pursuit of competitive grants, creating institutional inertia and limiting research development.

### Absence of a national health research policy

Although Honduras does not have a formal national health research policy, several broader initiatives indirectly influenced the research environment during the study period. In 2010, the government approved the National Science, Technology, and Innovation Policy, which outlined general priorities for scientific development, though its implementation was limited by underfunding and institutional fragmentation. In 2015, the Ministry of Health launched the National Health Model, emphasizing primary care and surveillance; while not explicitly a research policy, it created opportunities for operational and applied research, particularly in epidemiology and infectious diseases. However, in the absence of a coherent policy framework, decision-makers facing emerging health challenges often rely on technical support from international agencies such as PAHO, CDC-CAR [67], SE-COMISCA [68] and CHAI [69], rather than locally generated evidence. Although donor proposals frequently reference ‘applied research,’ funding for evidence generation remains scarce. Weak collaboration between the Ministry of Health and academia, combined with the lack of a national research council or priority-setting mechanisms, has left efforts fragmented and largely donor-driven.

### Brain drain and limited career pathways

Stable scientific careers remain scarce. While some MSc and PhD graduates have secured posts at UNAH, private universities, or national laboratories, many face precarious contracts, limited funding, and unclear promotion pathways. This lack of incentives drives highly trained individuals to migrate or leave research altogether [11]. The absence of new academic postgraduate programs at UNAH, combined with a lack of scholarships, further discourages local training. Many candidates opt for fully funded programs abroad rather than pay high tuition costs in Honduras.

### Weak linkages with the private sector remain a potential challenge

Interaction between universities and industry remains limited, hindering applied research and innovation. In Honduras and much of the region, business-sector researchers are scarce [59], and systematic evidence on the effects of these weak linkages is lacking. Mechanisms for collaboration in health are underdeveloped, and regulatory barriers, low trust, and the absence of fiscal incentives further discourage private R&D. Policy reforms and intermediary structures – such as innovation hubs and technology transfer offices – could help foster collaboration and innovation-led growth, but these strategies remain exploratory rather than proven solutions.

## Discussion and lessons learned

The Honduran experience over the past two decades illustrates the complex, non-linear process of building health research capacity in lower-middle-income countries. Despite persistent structural challenges, several enabling factors have sustained and expanded earlier gains. These lessons are relevant not only to Honduras but also to national actors, international donors, academic institutions, and research networks working in similarly fragile contexts.

A distinctive feature of the Canadian-funded initiative (2007–2012) was its explicit focus on health research capacity, institutional strengthening, and systemic change. This approach contrasted with earlier research capacity strengthening (RCS) models at UNAH – ranging from isolated training opportunities in high-income countries to the ‘sandwich model’ implemented by the KIRT program [70]. While these initiatives were highly beneficial, they often left to chance the research environment that foreign-trained scholars would encounter upon returning, limiting their ability to sustain long-term scientific activity. In contrast, the Canadian project combined individual training with deliberate institutional reforms, supporting the development of a Honduras-based graduate program designed and led by local faculty. This strategy embedded research training within national structures, creating opportunities for students unable to pursue training abroad and laying the groundwork for a more inclusive and sustainable research ecosystem.

The Canadian initiative adopted a multi-level model that combined actions at the individual, institutional, and systemic levels. At the individual level, it supported graduate training through the MSc program, mentoring, and participation in scientific writing, academic exchanges, and grant proposals. Institutionally, it fostered the development of laboratories, established UNAH’s first non-clinical Research Ethics Board, and strengthened faculty development. At the systemic level, it promoted inter-institutional collaboration, facilitated integration into regional networks, and encouraged alignment with international standards. This layered approach recognized that individual training is insufficient without robust institutional structures, and that institutional gains require broader policy alignment to achieve sustainability. A key pillar of the project was local ownership: Honduran faculty played central roles in curriculum design, laboratory management, ethical oversight, and network building, embedding these activities within UNAH’s governance systems and increasing their chances of long-term adoption. Rather than relying on temporary, project-dependent structures, the MSc program and the ethics board were formally incorporated into UNAH’s academic and administrative frameworks, ensuring continuity beyond the funding period. Sustainability was treated not as a byproduct but as a core design principle, supported by early planning, a gradual handover of responsibilities, and the cultivation of regional and national partnerships that reduced dependence on external funding and strengthened long-term viability.

## Future directions

UNAH’s experience over the last years highlights both the promise and fragility of research capacity in resource-limited settings. Consolidating and expanding these gains requires strategic actions at the institutional, national, and international levels:

### Institutional

Expanding researcher positions and granting administrative autonomy are essential to sustain capacity. At UNAH, limited understanding of research among many decision-makers has led to policies that hinder progress, such as rigid financial controls and lack of protected research time. Strengthening administrators’ skills in budgeting, legal, and research management is therefore critical. Structured dialogue between researchers and leadership has already supported more enabling regulations [71]. Effective fund administration is equally important: external project resources are managed through a centralized Foundation, but excessive oversight has caused delays that discourage competitive grants. Experience from the IIM shows that greater autonomy, paired with accountability, can improve efficiency. Simplifying procurement, authorizing multi-year implementation, and decentralizing hiring are critical reforms that require both regulatory adjustments and cultural shifts toward trust and shared responsibility.

### National

Establish a health research system and support early-career scientists. A key priority is the creation of a national health research system, modeled after Costa Rica’s INCIENSA [72] or INDICASAT in Panama [73]. Anchored within the Ministry of Health’s surveillance platform, this system could coordinate multidisciplinary research teams to address high-priority questions. The evidence generated should directly inform national disease control and elimination programs – including malaria elimination [74], the HIV/AIDS response [75], and dengue mortality reduction [76], aligned with global 2030 public health targets. Achieving this vision requires a governance framework that sets priorities secures sustained funding, regulates data, and upholds ethical standards. An autonomous or semi-autonomous body should coordinate academia, public health, and donors; without such leadership, efforts risk remaining fragmented and donor-driven. Supporting early-career scientists is equally important, through protected research time, seed funding, and mentorship [77]. Expanding the researcher model at UNAH, introducing tenure-track roles, and fostering interdisciplinary projects would help consolidate the next generation.

### International

The future of health research in Honduras also depends on stronger engagement with the private sector and international community. Collaboration in diagnostics, biotechnology, digital health, and pharmaceuticals remains limited, but with regulatory reforms, fiscal incentives, and innovation hubs, this gap could narrow. International cooperation, including South–South partnerships, is essential to secure funding, scientific platforms, and collaborative opportunities. A critical priority is simplifying importation of scientific supplies. Customs delays often compromise temperature-sensitive reagents; fast-track pathways for certified institutions would reduce waste, lower costs, and enable fuller participation in global science.

## Conclusion

Over the past decade, Honduras has shown that health research capacity can advance even amid national instability, scarce funding, and political uncertainty. Graduate programs, research laboratories, ethics boards, and a new generation of researchers mark a departure from the near absence of activity before 2012. Yet these gains are endangered. Chronic underinvestment, rigid administration, weak policies, and limited research careers pathways threaten sustainability. Still, international partnerships, diaspora scientists, institutional leadership, and regional networks have been vital enablers. Honduras illustrates that capacity building is a long-term, political, and iterative process requiring institutional reform, sustained investment, and strategic alliances. Its experience shows research capacity is not just a technical goal, but a strategy for equity, autonomy, and scientific resilience.

## Data Availability

No new data was generated in this study.

## References

[1] Sanchez AL, Canales M, Enriquez L, et al. A research capacity strengthening project for infectious diseases in Honduras: experience and lessons learned. Glob Health Action. 2013;6:21643. doi: 10.3402/gha.v6i0.21643 Epub 20130807; PubMed PMID: 23930937; PubMed Central PMCID: PMC3739968. 23930937 PMC3739968

[2] RICYT. El estado de la ciencia. Principales indicadores de ciencia y tecnología iberoamericanos/interamericanos 2020. Montevideo: UNESCO, Oficina Regional de Ciencias para América Latina y el Caribe; 2020. [cited 2025 Aug 19]. Available from: https://www.ricyt.org/en/2020/11/1617/

[3] Ong M. A comprehensive framework identifying barriers to global health R&D innovation and access. BMJ Glob Health. 2023;8:e013076. doi: 10.1136/bmjgh-2023-013076 Epub e013076; PubMed PMID: 37751936; PubMed Central PMCID: PMC10533694. PMC10533694 37751936

[4] Zhao L, Zhao Y, Du J, et al. Mapping the research on health policy and services in the last decade (2009–2018): a bibliometric analysis. Front Public Health. 2022;10:773668. doi: 10.3389/fpubh.2022.773668 Epub 20220427; PubMed PMID: 35570893; PubMed Central PMCID: PMC9092023. 35570893 PMC9092023

[5] Scopus. Scopus: a comprehensive abstract and citation database for impact makers. Elsevier; 2025. [cited 2025 Aug 23]. Available from: https://www.scopus.com/home.uri

[6] World Bank Group. GDP per capita (current US$). Central America: World Bank; 2025. [cited 2025 Sep 19]. Available from: https://data.worldbank.org/indicator/NY.GDP.PCAP.CD?end=2024&locations=L6&start=2000&utm_source=chatgpt.com&view=chart

[7] World Bank Group. Research & development expenditure (% of GDP): world Bank. 2025. [2025 Sep 19]. Available from: https://data.worldbank.org/indicator/GB.XPD.RSDV.GD.ZS

[8] World Bank Group. Researchers in R&D (per million people) 2025. [2025 Sep 19]. Available from: https://data.worldbank.org/indicator/SP.POP.SCIE.RD.P6

[9] PAHO. Health in the Americas: 2012 edition: regional outlook and country profiles. (WA) D.C.: PAHO; 2012. [cited 2025 Aug 19]. Available from: https://hia.paho.org/sites/default/files/2021-05/hia-2012-en.pdf

[10] Jarquin-Solis ME, Mauduit JC. Institutional capacity for science diplomacy in Central America. Front Res Metr Anal. 2021;6:663827. doi: 10.3389/frma.2021.663827 Epub 20210714; PubMed PMID: 34337309; PubMed Central PMCID: PMC8317690. 34337309 PMC8317690

[11] Ciocca DR, Delgado G. The reality of scientific research in Latin America; an insider’s perspective. Cell Stress Chaperones. 2017;22:847–11. doi: 10.1007/s12192-017-0815-8 PubMed PMID: 28584930; PubMed Central PMCID: PMC5655372. 28584930 PMC5655372

[12] International development cooperation strategies from Ministry for Foreign Affairs. Phase-out strategy for Swedish support to Honduras, January 2008–December 2011 2010. [Updated Published 01 March 2010, Updated 17 May 2015, cited 2025 August 11th]. Available from: https://www.government.se/international-development-cooperation-strategies/2010/03/phase-out-strategy-for-swedish-support-to-honduras-january-2008—december-2011/

[13] Sanchez AL, Canales M, Enriquez L, et al. Research capacity strengthening in Honduras. Lancet Glob Health. 2013;1:e75. doi: 10.1016/S2214-109X(13)70033-0 Epub 20130724; PubMed PMID: 25104159. 25104159

[14] DAAD. Daad Germany: DAAD. 2025. [cited 2025 Aug 16]. Available from: https://www.daad.de/en/

[15] IIM. Instituto de Investigaciones en Microbiología Tegucigalpa. [cited 2025 Aug 11]. Available from: https://iim.unah.edu.hn

[16] Joyce AL, Moreno M, Palomo L, et al. Genetic variability of Aedes aegypti (Diptera: Culicidae) in El Salvador and Honduras: presence of a widespread haplotype and implications for mosquito control. Parasit Vectors. 2024;17:229. doi: 10.1186/s13071-024-06312-7 Epub 20240516; PubMed PMID: 38755689; PubMed Central PMCID: PMC11100194. 38755689 PMC11100194

[17] Manabe YC, Betz J, Jackson O, et al. Clinical evaluation of the BioFire global fever panel for the identification of malaria, leptospirosis, chikungunya, and dengue from whole blood: a prospective, multicentre, cross-sectional diagnostic accuracy study. Lancet Infect Dis. 2022;22:1356–1364. doi: 10.1016/S1473-3099(22)00290-0 Epub 20220615; PubMed PMID: 35716700; PubMed Central PMCID: PMC9420791. 35716700 PMC9420791

[18] Reyes-Perdomo C, Escobar D, Galo L, et al. Insecticide resistance status and high frequency of kdr mutations in Aedes aegypti in Tegucigalpa, Honduras. Parasit Vectors. 2025;18:321. doi: 10.1186/s13071-025-06953-2 Epub 20250801; PubMed PMID: 40751217; PubMed Central PMCID: PMC12317443. 40751217 PMC12317443

[19] Avalos S, Mejia RE, Banegas E, et al. G6PD deficiency, primaquine treatment, and risk of haemolysis in malaria-infected patients. Malar J. 2018;17:415. doi: 10.1186/s12936-018-2564-2 Epub 20181108; PubMed PMID: 30409136; PubMed Central PMCID: PMC6225638. 30409136 PMC6225638

[20] Banegas S, Escobar D, Pinto A, et al. Asymptomatic malaria reservoirs in Honduras: a challenge for elimination. Pathogens. 2024;13:541. Epub 20240627. doi: 10.3390/pathogens13070541 PubMed PMID: 39057768; PubMed Central PMCID: PMC11280452. 39057768 PMC11280452

[21] Escobar D, Archaga O, Reyes A, et al. A follow-up to the geographical distribution of Anopheles species in malaria-endemic and non-endemic areas of Honduras. Insects. 2022;13:548. doi: 10.3390/insects13060548 Epub 20220615; PubMed PMID: 35735885; PubMed Central PMCID: PMC9225189. 35735885 PMC9225189

[22] Escobar D, Ascencio K, Ortiz A, et al. Distribution and phylogenetic diversity of Anopheles species in malaria endemic areas of Honduras in an elimination setting. Parasit Vectors. 2020;13:333. doi: 10.1186/s13071-020-04203-1 Epub 20200701; PubMed PMID: 32611432; PubMed Central PMCID: PMC7329488. 32611432 PMC7329488

[23] Escobar D, Ascencio K, Ortiz A, et al. Blood meal sources of Anopheles spp. in malaria endemic areas of Honduras. Insects. 2020;11:450. doi: 10.3390/insects11070450 Epub 20200716; PubMed PMID: 32708582; PubMed Central PMCID: PMC7412045. 32708582 PMC7412045

[24] Escobar D, Perez F, Ortiz B, et al. Pcr-rflp assays for the identification of Anopheles (Diptera: Culicidae) species circulating in Honduras. Malar J. 2023;22:57. doi: 10.1186/s12936-023-04494-6 Epub 20230218; PubMed PMID: 36805673; PubMed Central PMCID: PMC9938605. 36805673 PMC9938605

[25] Fontecha G, Escobar D, Ortiz B, et al. A PCR-RFLP technique to assess the geographic origin of Plasmodium falciparum strains in Central America. Trop Med Infect Dis. 2022;7:149. doi: 10.3390/tropicalmed7080149 Epub 20220726; PubMed PMID: 35893657; PubMed Central PMCID: PMC9394469. 35893657 PMC9394469

[26] Fontecha G, Escobar D, Ortiz B, et al. Field evaluation of a hemozoin-based malaria diagnostic device in Puerto Lempira, Honduras. Diagn (Basel). 2022;12:1206. doi: 10.3390/diagnostics12051206 Epub 20220511; PubMed PMID: 35626361; PubMed Central PMCID: PMC9140950. PMC9140950 35626361

[27] Fontecha G, Mejia RE, Banegas E, et al. Deletions of pfhrp2 and pfhrp3 genes of Plasmodium falciparum from Honduras, Guatemala and Nicaragua. Malar J. 2018;17:320. doi: 10.1186/s12936-018-2470-7 Epub 20180831; PubMed PMID: 30170596; PubMed Central PMCID: PMC6119307. 30170596 PMC6119307

[28] Fontecha G, Pinto A, Archaga O, et al. Assessment of Plasmodium falciparum anti-malarial drug resistance markers in pfcrt and pfmdr1 genes in isolates from Honduras and Nicaragua, 2018–2021. Malar J. 2021;20:465. doi: 10.1186/s12936-021-03977-8 Epub 20211214; PubMed PMID: 34906144; PubMed Central PMCID: PMC8670165. 34906144 PMC8670165

[29] Fontecha G, Pinto A, Escobar D, et al. Genetic variability of Plasmodium falciparum histidine-rich proteins 2 and 3 in Central America. Malar J. 2019;18:31. doi: 10.1186/s12936-019-2668-3 Epub 20190131; PubMed PMID: 30704496; PubMed Central PMCID: PMC6357481. 30704496 PMC6357481

[30] Matamoros G, Escobar D, Pinto A, et al. PET-PCR reveals low parasitaemia and submicroscopic malarial infections in Honduran Moskitia. Malar J. 2023;22:110. doi: 10.1186/s12936-023-04538-x Epub 20230328; PubMed PMID: 36978056; PubMed Central PMCID: PMC10053754. 36978056 PMC10053754

[31] Paz S, Escobar D, Fontecha G. Update on the susceptibility to chloroquine of Plasmodium falciparum in Honduras. Travel Med Infect Dis. 2023;54:102600. doi: 10.1016/j.tmaid.2023.102600 Epub 20230612; PubMed PMID: 37315826. 37315826

[32] Pinto A, Archaga O, Mejia A, et al. Evidence of a recent bottleneck in Plasmodium falciparum populations on the Honduran-Nicaraguan border. Pathogens. 2021;10:1432. doi: 10.3390/pathogens10111432 Epub 20211104; PubMed PMID: 34832588; PubMed Central PMCID: PMC8617645. 34832588 PMC8617645

[33] Zamora A, Pinto A, Escobar D, et al. Genetic diversity of Plasmodium vivax and Plasmodium falciparum field isolates from Honduras in the malaria elimination phase. Curr Res Parasitol Vector Borne Dis. 2025;7:100230. doi: 10.1016/j.crpvbd.2024.100230 Epub 20241121; PubMed PMID: 39759387; PubMed Central PMCID: PMC11699087. 39759387 PMC11699087

[34] Araujo Flores GV, Sandoval Pacheco CM, Tomokane TY, et al. Infectivity studies of Leishmania (Leishmania) infantum chagasi isolated from non-ulcerated cutaneous leishmaniasis. Rev Inst Med Trop Sao Paulo. 2025;67:e21. doi: 10.1590/S1678-9946202567021 Epub 20250404; PubMed PMID: 40197964; PubMed Central PMCID: PMC11981080. 40197964 PMC11981080

[35] Batista LFS, Sandoval Pacheco CM, Flores GVA, et al. Molecular insights into cell-mediated immunity in atypical non-ulcerated cutaneous leishmaniasis. Microorganisms. 2025;13:413. doi: 10.3390/microorganisms13020413 Epub 20250213; PubMed PMID: 40005779; PubMed Central PMCID: PMC11858551. 40005779 PMC11858551

[36] Silveira FT, Flores GVA, Pacheco CMS, et al. A comprehensive phenotypic and genotypic taxonomic review of Leishmania (Leishmania) poncei n. sp. (Kinetoplastea: Trypanosomatidae): a novel agent of cutaneous (non-ulcerated) and visceral leishmaniasis in Honduras, Central America. Trop Dis Travel Med Vaccines. 2025;11:27. doi: 10.1186/s40794-025-00264-1 Epub 20250728; PubMed PMID: 40717096; PubMed Central PMCID: PMC12302823. 40717096 PMC12302823

[37] Silveira FT, Sousa Junior EC, Silvestre RV, et al. Whole-genome sequencing of Leishmania infantum chagasi isolates from Honduras and Brazil. Microbiol Resour Announc. 2021;10:e0047121. doi: 10.1128/MRA.00471-21 Epub 20211202; PubMed PMID: 34854707; PubMed Central PMCID: PMC8638571. 34854707 PMC8638571

[38] Silveira FT, Sousa Junior EC, Silvestre RVD, et al. Comparative genomic analyses of new and old world viscerotropic Leishmanine parasites: further insights into the origins of visceral leishmaniasis agents. Microorganisms. 2022;11:25. doi: 10.3390/microorganisms11010025 Epub 20221221; PubMed PMID: 36677318; PubMed Central PMCID: PMC9865424. 36677318 PMC9865424

[39] Sosa-Ochoa W, Varela Amador J, Lozano-Sardaneta Y, et al. Detection of Leishmania infantum DNA in Pintomyia evansi and Lutzomyia longipalpis in Honduras. Parasit Vectors. 2020;13:593. doi: 10.1186/s13071-020-04462-y Epub 20201123; PubMed PMID: 33228800; PubMed Central PMCID: PMC7684752. 33228800 PMC7684752

[40] Sosa-Ochoa W, Zuniga C, Chaves LF, et al. Clinical and immunological features of human leishmania (L.) infantum-infection, novel insights Honduras, Central America. Pathogens. 2020;9:554. doi: 10.3390/pathogens9070554 Epub 20200710; PubMed PMID: 32664223; PubMed Central PMCID: PMC7399949. 32664223 PMC7399949

[41] Sosa-Ochoa W, Zuniga C, Flores GVA, et al. Cohort study of human infection by Leishmania (L.) infantum chagasi in southern Honduras, Central America. Trans R Soc Trop Med Hyg. 2025.:traf077. doi: 10.1093/trstmh/traf077 Epub 20250717; PubMed PMID: 40673876. 40673876

[42] Matamoros G, Rueda MM, Rodriguez C, et al. High endemicity of soil-transmitted helminths in a population frequently exposed to albendazole but no evidence of antiparasitic resistance. Trop Med Infect Dis. 2019;4:73. doi: 10.3390/tropicalmed4020073 Epub 20190427; PubMed PMID: 31035610; PubMed Central PMCID: PMC6631243. 31035610 PMC6631243

[43] Palma A, Ortiz B, Mendoza L, et al. Molecular analysis of human- and pig-derived Ascaris in Honduras. J Helminthol. 2019;93:154–158. doi: 10.1017/S0022149X18000160 Epub 20180305; PubMed PMID: 29502555. 29502555

[44] Wang AG, Dong T, Mansour H, et al. Paper-based DNA reader for visualized quantification of soil-transmitted helminth infections. ACS Sens. 2018;3:205–210. doi: 10.1021/acssensors.7b00857 Epub 20180116; PubMed PMID: 29336569. 29336569

[45] Doyle SR, Soe MJ, Nejsum P, et al. Population genomics of ancient and modern Trichuris trichiura. Nat Commun. 2022;13:3888. doi: 10.1038/s41467-022-31487-x Epub 20220706; PubMed PMID: 35794092; PubMed Central PMCID: PMC9259628. 35794092 PMC9259628

[46] Matamoros G, Sanchez A, Cimino R, et al. A comparison of the diagnostic capability of Kato-Katz and real-time PCR for the assessment of treatment efficacy of ivermectin and albendazole combination against T. trichiura infections. PLOS Negl Trop Dis. 2024;18:e0012677. doi: 10.1371/journal.pntd.0012677 Epub 20241119; PubMed PMID: 39561184; PubMed Central PMCID: PMC11614246. 39561184 PMC11614246

[47] Matamoros G, Sanchez A, Gabrie JA, et al. Efficacy and safety of albendazole and high-dose ivermectin coadministration in school-aged children infected with Trichuris trichiura in Honduras: a randomized controlled trial. Clin Infect Dis. 2021;73:1203–1210. doi: 10.1093/cid/ciab365 PubMed PMID: 33906234. 33906234

[48] Naceanceno KS, Matamoros G, Gabrie JA, et al. Use of multi-parallel real-time quantitative PCR to determine Blastocystis prevalence and association with other gastrointestinal parasite infection in a rural Honduran location. Am J Trop Med Hyg. 2020;102:1373–1375. doi: 10.4269/ajtmh.19-0876 PubMed PMID: 32189609; PubMed Central PMCID: PMC7253141. 32189609 PMC7253141

[49] Fontecha G, Montes K, Ortiz B, et al. Identification of cryptic species of four Candida complexes in a culture collection. J Fungi (Basel). 2019;5:117. doi: 10.3390/jof5040117 Epub 20191217; PubMed PMID: 31861048; PubMed Central PMCID: PMC6958398. 31861048 PMC6958398

[50] Fontecha G, Sanchez A, Ortiz B. Publication trends in neglected tropical diseases of Latin America and the Caribbean: a bibliometric analysis. Pathogens. 2021;10:356. doi: 10.3390/pathogens10030356 Epub 20210317; PubMed PMID: 33802834; PubMed Central PMCID: PMC8002643. 33802834 PMC8002643

[51] Montes K, Ortiz B, Galindo C, et al. Identification of Candida species from clinical samples in a Honduran tertiary hospital. Pathogens. 2019;8:237. doi: 10.3390/pathogens8040237 Epub 20191115; PubMed PMID: 31731617; PubMed Central PMCID: PMC6963973. 31731617 PMC6963973

[52] Ortiz B, Aguilar K, Galindo C, et al. Candida species isolated from clinical samples in a tertiary hospital in Honduras: where is Candida auris? Curr Med Mycol. 2022;8:1–8. doi: 10.18502/cmm.8.3.11212 PubMed PMID: 37051554; PubMed Central PMCID: PMC10084484. PMC10084484 37051554

[53] Ortiz B, Aguilar K, Luque M, et al. Mixed candidemia in a pediatric patient with Hirschsprung’s disease. Rev Iberoam Micol. 2023;40:15–16. doi: 10.1016/j.riam.2022.12.003 Epub 20230226; PubMed PMID: 36849320. 36849320

[54] Ortiz B, Ballesteros-Monrreal MG, Rosales-Tamashiro J, et al. Global insights and trends in research on dermatophytes and dermatophytosis: a bibliometric analysis. Mycoses. 2024;67:e13803. doi: 10.1111/myc.13803 PubMed PMID: 39343727. 39343727

[55] Ortiz B, Lainez-Arteaga I, Galindo-Morales C, et al. First molecular identification of three clinical isolates of fungi causing mucormycosis in Honduras. Infect Dis Rep. 2022;14:258–265. doi: 10.3390/idr14020031 Epub 20220407; PubMed PMID: 35447883; PubMed Central PMCID: PMC9027499. 35447883 PMC9027499

[56] Ortiz B, Lopez R, Munoz C, et al. First report of the emerging pathogen Kodamaea ohmeri in Honduras. J Fungi (basel). 2024;10:186. doi: 10.3390/jof10030186 Epub 20240228; PubMed PMID: 38535195; PubMed Central PMCID: PMC10971700. 38535195 PMC10971700

[57] Ortiz B, Perez-Aleman E, Galo C, et al. Molecular identification of Candida species from urinary infections in Honduras. Rev Iberoam Micol. 2018;35:73–77. doi: 10.1016/j.riam.2017.07.003 Epub 20180213; PubMed PMID: 29395833. 29395833

[58] Ortiz B, Varela D, Fontecha G, et al. Strengthening fungal infection diagnosis and treatment: an in-depth analysis of capabilities in Honduras. Open Forum Infect Dis. 2024;11:ofae578. doi: 10.1093/ofid/ofae578 Epub 20241003; PubMed PMID: 39421702; PubMed Central PMCID: PMC11483579. 39421702 PMC11483579

[59] UNESCO. UNESCO science report 2021 Paris, FR: UNESCO. 2021. [cited 2025 Aug 16]. Available from: https://www.unesco.org/reports/science/2021/en/latin-america

[60] UNESCO. Domestic spending on research and development as a share of GDP, 1996–2018. (%): Unesco; 2021. [cited 2025 Aug 23]. Available from: https://www.unesco.org/reports/science/2021/en/dataviz/research-spending-share-gdp

[61] World Bank Group. Research and development expenditure (% of GDP) 2024. Available from: https://data.worldbank.org/indicator/GB.XPD.RSDV.GD.ZS?utm

[62] Huete-Perez JA, Hernandez-Mondragon AC, Massey DS, et al. Catalyzing sustainable development: insights from the international workshop on STI policies and innovation systems in Central America. Front Res Metr Anal. 2024;9:1511393. doi: 10.3389/frma.2024.1511393 Epub 20241211; PubMed PMID: 39722835; PubMed Central PMCID: PMC11669327. 39722835 PMC11669327

[63] Instituto Conmemorativo Gorgas de Estudios de la Salud (ICGES). Gorgas: instituto Gorgas. 2025. [cited 2025 Aug 16]. Available from: https://www.gorgas.gob.pa

[64] Gorgas IC. Proyecto campus Gorgas Ciudad Panama 2025. [cited 2025 Aug]. Available from: https://www.gorgas.gob.pa/antecedentes-campus-gorgas/

[65] SENACYT. Secretaría Nacional de Ciencia, Tecnología e Innovación. Panama City: SENACYT; 2025. [cited 2025 Aug 19]. Available from: https://www.senacyt.gob.pa

[66] Secretaría de Finanzas G. Informe de seguimiento y evaluación de la ejecución presupuestaria física y financiera al segundo trimestre 2025. Tegucigalpa, Honduras: Gobierno de Honduras; 2025. [cited 2025 Aug 19]. Available from: https://www.sefin.gob.hn/wp-content/uploads/2025/presupuesto/02-trim/html/index_html_files/Evaluacion515.pdf

[67] Center for Global Health (U.S.). CDC in Central America: February 2020: CDC. 2025. [cited 2025 Aug 19]. Available from: https://stacks.cdc.gov/view/cdc/88848

[68] SICA. Se-Comisca y CDC desarrollan primer módulo de la quinta cohorte del nivel intermedio del FETP en Honduras 2025. Available from: https://www.sica.int/noticias/se-comisca-y-cdc-desarrollan-primer-modulo-de-la-quinta-cohorte-del-nivel-intermedio-del-fetp-en-honduras_1_130182.html

[69] Clinton Health Action Initiative. Chai: chai. 2025. [cited 2025 Aug 19]. Available from: https://www.clintonhealthaccess.org

[70] Moreno E, Gutierrez JM, Chaves-Olarte E. The struggle of neglected scientific groups: ten years of NeTropica efforts to promote research in tropical diseases in Central America. PLoS Negl Trop Dis. 2011;5:e1055. doi: 10.1371/journal.pntd.0001055 Epub 20110726; PubMed PMID: 21814583; PubMed Central PMCID: PMC3144179. 21814583 PMC3144179

[71] Ferrández-Berrueco RM, Moliner O, Sánchez-Tarazaga L, et al. University responsible research and innovation and society: dialogue or monologue? J Responsible Innov. 2023;10:1. doi: 10.1080/23299460.2023.2272331

[72] INCIENSA. Inciensa: instituto Costarricense de Investigación y Enseñanza en Nutrición y Salud: inciensa. 2025. [cited 2025 Aug 16]. Available from: https://www.inciensa.sa.cr

[73] INDICASAT-AIP. Indicasat-aip Panama City: indicasat. 2025. [cited 2025 Aug 16]. Available from: https://indicasat.org.pa

[74] WHO. Global technical strategy for malaria 2016–2030. Geneva: WHO; 2021. update [cited 2025 Aug 16]. Available from: https://iris.who.int/bitstream/handle/10665/342995/9789240031357-eng.pdf?sequence=1

[75] WHO. Global health sector strategies on, respectively, HIV, viral hepatitis and sexually transmitted infections for the period 2022–2030. Geneva: WHO; 2022. Available from: https://cdn.who.int/media/docs/default-source/hq-hiv-hepatitis-and-stis-library/full-final-who-ghss-hiv-vh-sti_1-june2022.pdf?sfvrsn=7c074b36_13

[76] WHO. Ending the neglect to attain the sustainable development goals: a road map for neglected tropical diseases 2021–2030: overview. WHO; 2020. [cited 2025 Aug 16]. Available from: https://iris.who.int/handle/10665/332094

[77] Yin HL, Gabrilove J, Jackson R, et al. Sustaining the clinical and translational research workforce: training and empowering the next generation of investigators. Acad Med. 2015;90:861–865. doi: 10.1097/ACM.0000000000000758 PubMed PMID: 26414054; PubMed Central PMCID: PMC4587496. 26414054 PMC4587496

